# Fatsia Japonica-Derived Hierarchical Porous Carbon for Supercapacitors With High Energy Density and Long Cycle Life

**DOI:** 10.3389/fchem.2020.00089

**Published:** 2020-02-20

**Authors:** Huiling Li, Lihua Cao, Feng Wang, Gaigai Duan, Wenhui Xu, Changtong Mei, Guoying Zhang, Kunming Liu, Meng Yang, Shaohua Jiang

**Affiliations:** ^1^Co-Innovation Center of Efficient Processing and Utilization of Forest Resources, College of Materials Science and Engineering, Nanjing Forestry University, Nanjing, China; ^2^School of Pharmacy, Jiangxi University of Traditional Chinese Medicine, Nanchang, China; ^3^Shangdong Key Laboratory of Biochemical Analysis, Qingdao, China; ^4^College of Chemistry and Molecular Engineering, Qingdao University of Science and Technology, Qingdao, China; ^5^School of Metallurgical and Chemical Engineering, Jiangxi University of Science and Technology, Ganzhou, China; ^6^Xiangyang Environmental Protection Monitoring Station, Xiangyang, China

**Keywords:** supercapacitors, hierarchical porous carbon, biomass materials, energy density, long cycle life

## Abstract

Fatsia Japonica seed, which is mainly composed of glucose, has potential as a porous carbon matrix precursor for supercapacitors that can achieve high-value utilization. Cost-effective hierarchical porous carbon materials (HPC) were prepared from Fatsia Japonica by annealing at high temperature. The pore size and distribution of the HPC can be precisely controlled and adjusted by altering the activation temperature. The HPC obtained at 600°C showed favorable features for electrochemical energy storage, with a surface area of 870.3 m^2^/g. The HPC for supercapacitors (a three-electrode system) exhibited good specific capacitance of 140 F/g at a current density of 1 A/g and a long cycling life stability (87.5% remained after 10,000 cycles). In addition, the HPC electrode showed an excellent energy density of 23 Wh/Kg. Such hierarchical porous biomass-derived carbon would be a good candidate for application in the electrodes of supercapacitors due to its simple preparation process and the outstanding electrochemical performance.

## Introduction

With the development of modern social science and technology and the increasing energy demand for power, a new generation of energy devices with advanced, low cost, and sustainable sources have attracted great attention from industry, including supercapacitors (SCs), Li-ion batteries (LIBs), and fuel cells (Shao et al., 2018; Ma et al., 2019; Lei et al., accepted). SCs have been considered as one of the most promising energy storage devices in the last decade for applications in portable electronic devices, vehicles, etc. (Han et al., 2019d; Wang et al., 2019). Due to their high energy density, long cycle life, and fast discharge/charge characteristics, SCs bridge the gap between conventional electrolytic capacitors and LIBs. Based on their energy storage mechanism, supercapacitors can be divided into two categories: electric double-layer capacitors (EDLC) and pseudo-capacitors (Choi et al., 2019; Zhao et al., 2019b). However, the key to the electrochemical performance of SCs lies in the choice and design of electrode materials. Recently, porous carbonaceous materials have been widely commercialized as active materials for SCs on the basis of their controllable porosity, high specific surface area, and electrochemical stability, but the dramatic drawback of commercial EDLC is a relatively low energy density in the range of 5–10 Wh/Kg (Borenstein et al., 2017; Zhao et al., 2019a; Wang et al., 2020).

Over the years, much effort has been applied toward improving the energy density of carbonaceous materials, such as facilitating a controllable hierarchical porous structure and designing the nanostructure to improve specific surface area and ion transport (Li and Wei, 2013; Benzigar et al., 2018) and doping heteroatoms (N, S, P, etc.) to introduce active reaction sites (Chen et al., 2019; Yan et al., 2019). The most fascinating work is the heteroatom-doping through template method, which can form structural defects on the surface of carbon material to increase conductivity and improve wettability (Huijuan et al., 2017; Li et al., 2017). Na et al. (2017) fabricated nitrogen and fluorine-doped mesoporous carbon nanofibers (NFMCNFs) by the hydrothermal method and a subsequent vacuum plasma process. The NFMCNF electrode exhibited a high specific capacitance of 252.6 F/g at a current density of 0.5 A/g. Lv et al. (2018) prepared N and P co-doped carbon hollow spheres (NPCHSs) through a carbonization and subsequent chemical activation route. The NPCHSs present a high specific surface area of 1,155 m^2^/g due to their 3D connected porous structure and a high specific capacitance of 232 F/g at a current density of 1 A/g. Mao et al. (2017) reported N_2_-doped graphite (NG) as the negative electrode and a kind of mesoporous NiCo_2_O_4_ nanorod/graphene oxide (NiCo_2_O_4_/GO) composite as the positive electrode. The symmetric supercapacitor displayed high energy density of 34.3 Wh/Kg at a power density of 800 W/Kg. Thus, the prepared porous carbon materials with heteroatom- doping can obviously improve electrochemical performance. However, most active carbons (ACs) on improving electrochemical performance introduce heteroatoms by chemical and physical routes, which can result in high cost, environmentally destructive, and complicated manufacturing. Biomass and its derivatives, not only benefiting from renewable, low-cost, and environmentally friendly properties and but also from being rich in other elements such as nitrogen and oxygen, have been considered as prospective carbon precursors (Abioye and Ani, 2015; Lu et al., 2018; Hou et al., 2019). Many porous carbonaceous materials based on natural sources have been prepared, such as willow catkins (Wang et al., 2015), tea leaves (Song et al., 2019), corncob (Karnan et al., 2017), peanut shell (He et al., 2013), banana peels (Zhang et al., 2016), bamboo (Zequine et al., 2016), seaweed (Ye et al., 2018), biomass-based composites (Sun et al., 2017; Han et al., 2019a) etc., which show good electrochemical performance for EDCL.

Fatsia Japonica, a subtropical species, is native to southern Japan as well as southern China. The plants are commonly used as a graceful ornamental tree and have potential medicinal value (Luo et al., 2012; Shi et al., 2017). The seeds, appearing from October to May of the next year, have a long maintenance period and are plentiful in the tree, so they can be picked at any time. Little research on the composition of Fatsia Japonica has been reported (Ye et al., 2014). Aokia et al. (1981) have analyzed the chemical constituents of the essential oils in the stems, leaves, and fruits of the Fatsia Japonica. A total of 97 compounds were identified in the essential oils extracted from the roots, leaves, and fruits of the Fatsia Japonica, mainly including monoterpenoids and their oxides and semiquinones and their oxides. Thus, the seeds, with have potential as medicines, are rich in other elements (oxygen etc.) besides carbon, which could result in a decrease in cyclic stability due to the provision of a reacting active site. However, biomass material with more oxygen atoms has a self-doping effect that improves the electrochemical performance and wettability of carbon materials. Moreover, the seeds have strong solution absorption capacity due to their macropore structure, which provides an excellent platform for further optimizing their structure and properties (Kil et al., 2008). In order to realize transformation into higher-value products, we induced the seeds to become carbonized under low temperature in our preliminary work. We can observe from scanning electron microscopy (SEM) that the carbon materials obtained possess many macropores, which can be easily controlled to form a hierarchical porous structure. Herein, the aim of our research work is to exploit a novel biomass material with a controllable pore distribution and enrich the choice of precursors for electrode materials of EDLC.

In this work, a facile method involving pre-carbonization at low temperature and subsequent pyrolysis and activation with KOH at high temperatures was developed to fabricate hierarchical porous carbon materials (HPCs) derived from Fatsia Japonica. The HPCs obtained showed remarkable features of good conductivity, high energy density, and promising electrochemical properties. The relationships between the structural characteristics, activation temperatures, and electrochemical performance were investigated intensively.

## Materials

The seeds of Fatsia Japonica were obtained from the trees around our laboratory (Nanjing China). All other chemicals were of reagent grade without further purification. Deionized water was used throughout the experiments.

### Preparation of Hierarchical Porous Carbon (HPC)

Prior to the synthesis of HPCs, the fresh seeds were first pretreated by soaking in aqueous HCl solution (1 M) for about 2 h, followed by washing with deionized water and oven drying at 60°C for 12 h. Subsequently, the dried seeds were pre-carbonized in a muffle furnace at 300°C to remove other organic substances thoroughly. The sample of pre-carbonized seeds obtained was named PCS. The mixture was then transferred to a crucible, followed by annealing and activating at the desired temperature for 12 h under an N_2_ atmosphere (Zhang and Chen, 2015; Hou et al., 2019). The temperature was raised to 300°C at a rate of 3°C/min, then at a rate of 5°C/min. To prepare various HPCs, different carbonization and activation temperatures (e.g., 500, 600, 700, and 800°C) were investigated, and the corresponding samples were designated as HPC-500, HPC-600, HPC-700, and HPC-800, respectively.

### Characterization of HPC Samples

Field emission scanning electron microscopy (FESEM) measurements were performed on a JEOL JSM-7001F microscope at an accelerating voltage of 10 kV to observe the morphologies and structures of the samples. The pore structure of the obtained samples was examined through nitrogen adsorption/desorption experiments at 77 K using a micromeritics apparatus (ASAP 2020 V3.02 H). The specific surface area was measured based on the Brunauer-Emmett-Teller (BET) method, and the BJH method was used to calculate the pore size distribution and pore volumes. Raman spectra were collected from a Raman spectrometer (Jobin Yvon, HR800). X-ray photoelectron spectroscopy (XPS) was performed on a KRATOS Axis Ultra photoelectron spectrometer using Al Kα radiation at a power of 225 W.

### Electrochemical Measurement

The electrochemical performance of the prepared HPCs was measured by using a three-electrode system in 6 M KOH aqueous electrolyte at room temperature. An Hg/HgO electrode (saturated in 1 M KOH solution) and platinum sheets were used as reference electrode and counter electrodes, respectively. The working electrode was prepared by pressing a slurry mixture of the obtained HPC (80 wt%), acetylene black (10 wt%), and polyvinylidene fluoride (PVDF, 10 wt%) onto a piece of Nickel foam and then dried at 60°C for 12 h. The surface area of the working electrode is about 1 cm^2^, and the mass loading of the active materials is about 2 mg/cm^2^. Cyclic voltammetry (CV), galvanostatic charge-discharge (GCD), and electrochemical impedance spectroscopy (EIS) tests were carried out on a CHI 600 electrochemical workstation (Shanghai Chenhua, China). The working voltages window was commonly between −1 and 0.1 V.

The specific capacitance in the three-electrode system was calculated from the GCD curves according to the following Equation (1). The energy density and power density were calculated using Equation (2) and Equation (3), respectively.

(1) $$\begin{array}{l} C=\frac{I^{*}\Delta t}{m^{*}\Delta V} \end{array}$$

(2) $$\begin{array}{l} E=\frac{1}{2}C^{*}\Delta V^{2} \end{array}$$

(3) $$\begin{array}{l} P=\frac{E}{\Delta }t \end{array}$$

where *C* is the specific capacitance (F/g), *I* is the discharge current (A), Δ*t* is the discharge time (s), *m* represents the mass of active material in the electrode (g), Δ*V* is the potential change in discharge (V), *E* is the energy density (Wh/Kg), and *P* is the power density (W/Kg).

The symmetric supercapacitors using two equal-power electrodes were assembled into a button battery system, and 6 M KOH was used as the electrolyte. The specific capacitance of symmetric supercapacitors was calculated by Equation (4).

(4) $$\begin{array}{l} C=\frac{4I\Delta t}{m\Delta V} \end{array}$$

where *m* (g) is the total mass of the active material.

## Results and Discussion

**Scheme 1:**
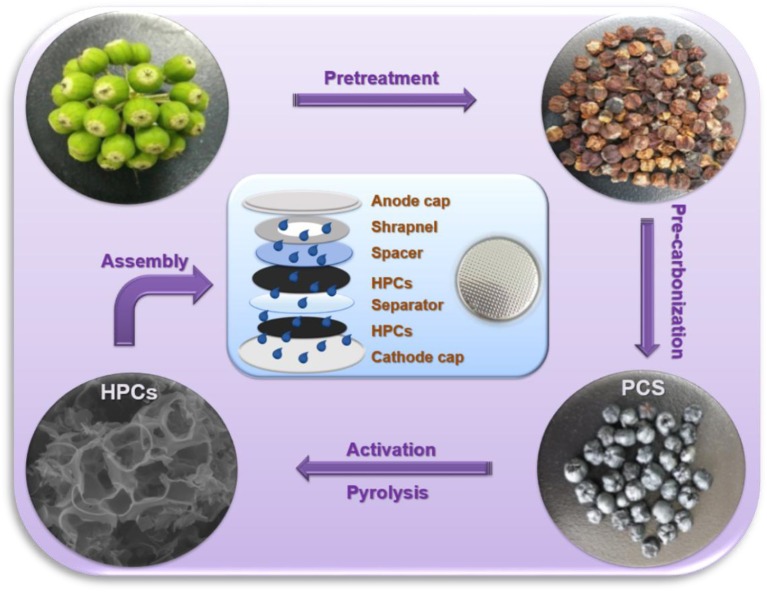
Schematic illustration of the fabrication process of HPCs.

The formation process of HPCs from the seeds of Fatsia Japonica is simply illustrated in Scheme 1. Briefly, the fresh seeds were pre-treated with hydrochloric acid (HCl) to remove the inorganic substances preliminarily and then pre-carbonized at a low temperature (300°C) to remove other organic substances. To further optimize the pore structure, pre-carbonized seeds were mixed with KOH and then carbonized at 500, 600, 700, and 800°C, respectively.

**Figure 1:**
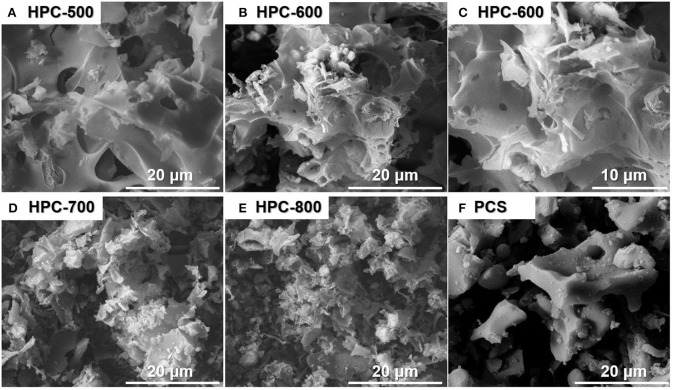
FESEM images of the HPCs prepared at different activation temperatures **(A–E)** and the PCS **(F)**.

The morphologies of HPCs fabricated at different activation temperatures and the PCS without activation are shown in Figure 1. Compared to the PCS sample (Figure 1F), the samples prepared by the activation process present a sheet-like structure rather than a bulk structure, which shows that the activation process contributes to the fabrication of a porous structure through the activation agent (KOH) etching the wall of the PCS sample with a macropore structure. Based on Figure 1, the activation temperature has a major effect on the morphologies and structures of the resultant samples. The samples with different activation temperatures present different degrees of etching by KOH activation. The sample activated at 500°C (Figure 1A) exhibits a continuous sheet-like structure. As the temperature rises, the flaky morphology of the samples varies from thinner to fragmented. Especially, the sample HPC-800 (Figure 1F) presents a fragmented structure, which is attributed to the strong etching of KOH in the walls of the macropores or the inside of the sample and causes the pores to be larger, breaking up the sheet structure (Figure 1E). However, HPC-600 presents a complete layered lamellar structure (Figures 1B,C). Compared to HPC-500 and HPC-700, the sample of HPC-600 exhibits a uniform 3D network structure, which is promising for electrolyte ion diffusion (Benzigar et al., 2018). Thus, the sample pyrolyzed at 600°C shows a uniformly connected lamellar structure, which can provide fast channels for ion diffusion during the charge and discharge process.

**Figure 2:**
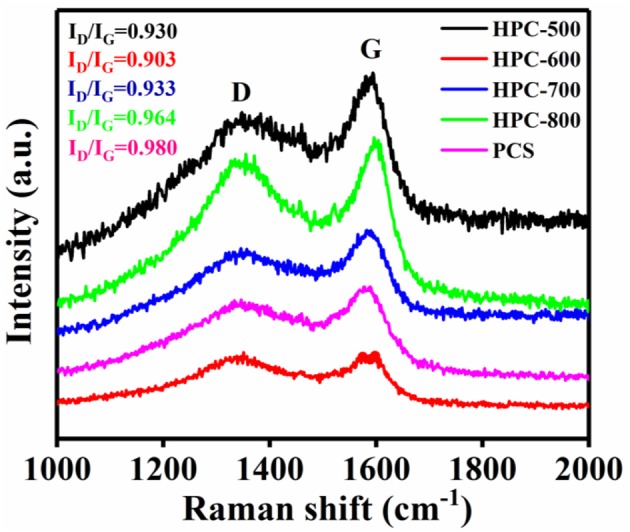
Raman spectra of the HPCs prepared at different temperatures.

Raman spectra of the samples are shown in Figure 2. Two obvious peaks located at 1,350 and 1,590 cm^−1^ for all the samples correspond to the D and G band, respectively. The G band is related to the degree of graphitization, while the D band is associated with local defects and disordered properties of HPCs (Zheng et al., 2017; He et al., 2019). The intensity ratio I_D_/I_G_ represents the degree of structural graphitization. A higher value means a lower degree of graphitization. The I_D_/I_G_ values of the samples were 0.930, 0.903, 0.933, 0.964, and 0.980, corresponding to HPC-500, HPC-600, HPC-700, HPC-800, and PCS, respectively. As the activation temperature increases from 500 to 600°C, the I_D_/I_G_ value increases, and it tends to decrease from 600 to 800°C. It is observed that the defect degree of the obtained HPC decreases and the degree of graphitization increases at 600°C. Thus, HPC-600 displays a higher degree of graphitization and lower defect degree, so it may possess good conductivity.

**Figure 3:**
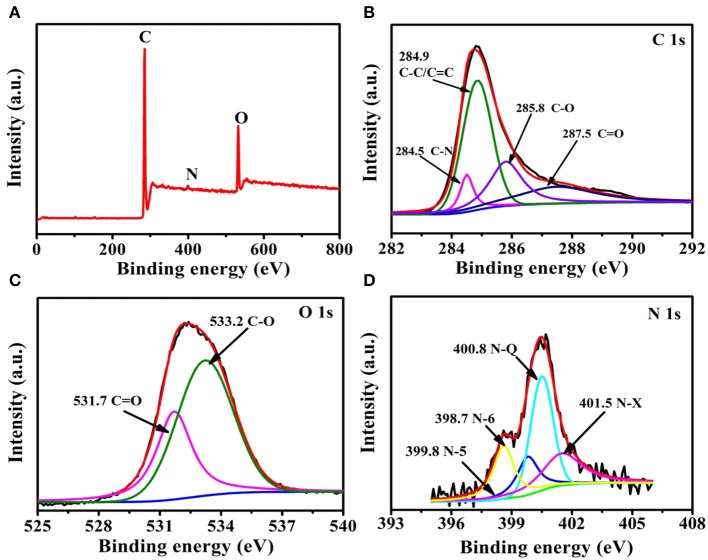
XPS spectra of HPC-600: **(A)** full energy spectrum, **(B)** C 1s, **(C)** O 1s, and **(D)** N 1 s.

Heteroatom doping is one of the common strategies for preparing high-performance supercapacitor carbon materials (Hou et al., 2018; Lee et al., 2018; Kim et al., 2019; Wu et al., 2019). Seeds are rich in a variety of active ingredients, so it is inferred that the biomass-derived carbon materials should have self-doped heteroatoms present within them. Thus, XPS (Figure 3) was carried out to study the surface chemical composition of the resulting sample. The full XPS spectra of HPC-600 derived from the seeds is shown in Figure 3A, from which C 1s, N 1s, and O 1s can be observed. The atomic percentages of C, N, and O were 82.4, 1.01, and 12.59%, respectively. The high-resolution spectrum of C 1s could be divided into four regions, which is credited to C-N (284.5 eV), C-C/C=C (284.9 eV), C-O (285.8 eV), and C=O (287.5 eV), respectively (Figure 3B). The N 1s (Figure 3C) spectrum reveals the presence of four nitrogen-based components, including pyridine nitrogen-oxide (N-X, 401.5 eV), graphitic nitrogen (N-Q, 400.8 eV), pyrrolic N (N-5, 399.8 eV), and pyridinic N (N-6, 398.7 eV). The O 1s spectrum was fitted to two individual peaks located at 531.7 and 533.2 eV, corresponding to C = O and C-O, respectively (Figure 3D). It can be speculated that the wettability of the prepared HPC could be improved due to heteroatoms (N, O) and high O atomic content (12.59%), which could contribute to the electrochemical performance of electrode materials.

**Figure 4:**
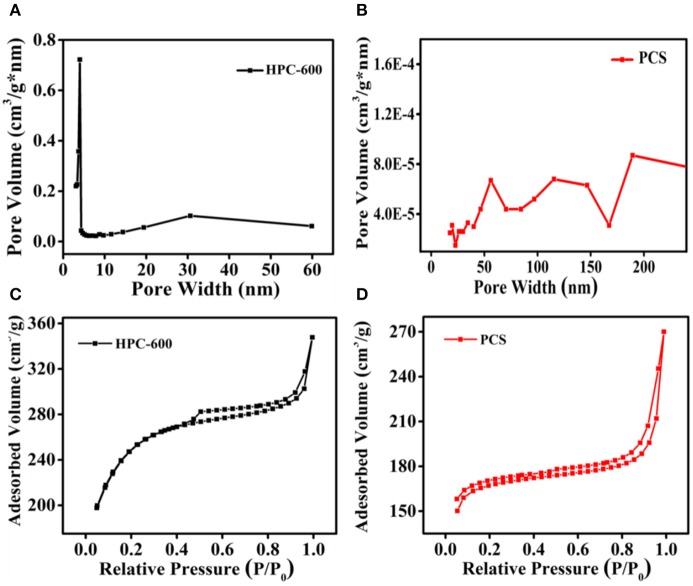
**(A,B)** Pore size distribution curves of HPC-600 and PCS, and **(C,D)** nitrogen adsorption/desorption isotherms of HPC-600 and PCS.

To further determine the relationship between morphology and porous structure and examine the formation of hierarchical pores, measurements by BET N_2_ adsorption/desorption technology were carried out; the results are depicted in Figure 4. According to the nitrogen adsorption and desorption isotherms (Figure 4A), the major sorption for the sample of HPC-600 occurs at a low relative pressure from 0.05 to 0.3 and exhibits hysteresis between adsorption and desorption, which is attributed to an obvious capillary phenomenon with the increase in relative pressure. Thus, the sample of HPC-600 shows the IV type nitrogen sorption isotherm, suggesting the existence of mesopores and macropores (Qu et al., 2015). However, the sample of PCS exhibits the II type nitrogen sorption isotherm and hysteresis at relative pressures from 0.01 to 0.8, which only demonstrate the emergence of weak gas-solid interaction. These findings are further supported by the pore size distribution (Figures 4C,D). The main pore widths for the sample of HPC-600 are about 2.2 nm and between 60 and 120 nm, which demonstrate the existence of smaller mesopores and macropores, respectively. For the sample of PCS, the pore size is distributed over macropore widths (> 50 nm), which is consistent with the observed nitrogen sorption isotherms. The specific surface area of HPC-600 (870.3 m^2^/g) was significantly higher than that of the PCS (510.6 m^2^/g), as were the pore volumes, which offers more contact area for electrolyte penetration. Ordering hierarchical pores can not only provide excellent accessibility to active sites and enhanced mass transport and diffusion during charge and discharge but also improve the specific surface area and electronic and ionic conductivity (Benzigar et al., 2018; Han et al., 2019c). Therefore, combined with the results of FESEM (Figure 1B), it can be concluded that HPC-600, with a 3D lamella-like structure, successfully possesses a hierarchical porous structure, which could accelerate the transport of electrons and diffusion of ions in electrolyte, improving the electrochemical performance.

The relationship between a hierarchical porous structure and electrochemical performance can be explained by the activation reaction. At high temperature, the KOH primarily penetrates inside the pore wall of the pre-carbide sample and reacts on the surface of carbon materials to form nanopores or mesopores. Products such as K_2_CO_3_ are then obtained and continually corrode inside the pre-carbide sample to form a more porous and three-dimensional connected porous structure (Lu et al., 2010; Zhang and Chen, 2015; Eftekhari, 2018). Herein, as the activation temperature raised, the more violent the reaction between KOH and carbon, and the larger the pore volume formed.

**Figure 5:**
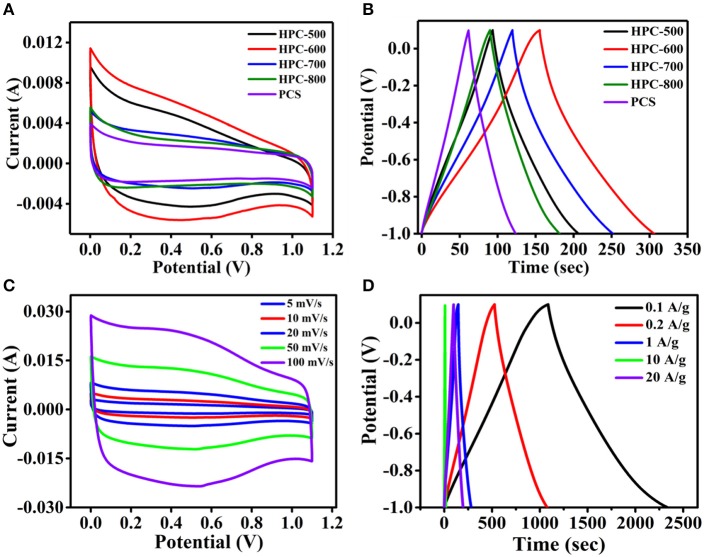
**(A)** CV curves of the samples HPC-500, HPC-600, HPC-700, HPC-800, and PCS at a scan rate of 10 mV/s; **(B)** GCD curves of HPCs at a current density of 1 A/g; **(C)** CV curves of HPC-600 at different scan rates; **(D)** GCD curves of the sample of HPC-600 at different current densities.

To evaluate the electrochemical performance of the hierarchical porous carbon, cyclic voltammetry (CV) and galvanostatic charge/discharge tests were carried out with a three-electrode configuration in an aqueous solution of 6 M KOH; the results are depicted in Figure 5. The CV curves of all samples are displayed from Figure 5A. One can see that all samples displayed a nearly rectangular shape at 10 mV/s, indicating the formation of an electric double layer and ideal capacitive behaviors. The results can be demonstrated from the GCD curves of all samples at the same current density (Figure 5B). Figure 5B shows that all samples present an equicrural quasi-triangle shape. However, based on the specific capacitance calculation in equation (3), the discharge time of sample HPC-600 was longer than those of the others, and the specific capacitance was about 140 F/g. This value of specific capacitance is larger than or at a similar level to other carbon materials, as summarized in Table 1. It is demonstrated that the sample of HPC-600 possesses better electrochemical performance, due to the evenly distributed mesopore structure and complete 3D lamella-like structure, which provide fast channels for easy ion diffusion in electrolyte.

**Table 1:** Comparison of electrochemical performance of carbon-based supercapacitors.

**Carbon type**	**Activating agent**	**Electrolyte type**	**Current density**	**Specific capacitance**	**References**
Banana fiber-derived carbon	ZnCl_2_	1 M Na_2_SO_4_	0.5 A/g	74 F/g	Sun and Sun, 2002
Oil palm kernel shell-based carbon	Steam activation	1 M KOH	0.5 A/g	123 F/g	Misnon et al., 2015
Corn stalk core	KOH	3 M KOH	1 A/g	140 F/g	Yu et al., 2018
Rice husk-derived carbon	H_3_PO_4_	1 M Na_2_SO_4_	1 A/g	112 F/g	Ganesan et al., 2014
MWCN/activated CNFs	NH_3_ steam	6 M KOH	0.5 A/g	160 F/g	Deng et al., 2013
PAN- and PVP-based CNF	None	0.5 H_2_SO_4_	0.2 A/g	104.5 F/g	Liu et al., 2015
PAN/PMMA-CFs	None	6 M KOH	1 A/g	140 F/g	Zhou et al., 2019
HPC	KOH	6 M KOH	1 A/g	140 F/g	This work

The capacitive performance of the hierarchical porous materials of HPC-600 was further measured with CV measurement at the same voltage window and GCD measurement at different current densities. Figure 5C depicts the CV curves of HPC-600. The HPC does not have faradic current effects during charge and discharge, and the sample presented a quasi-rectangular shape at different scan rates. In addition, as the scan rate increased, HPC-600 was still closer to a rectangular shape. It can be demonstrated that the HPC-600 exhibits excellent rate capability and good electrochemical behavior. This is further shown by the GCD measurement results in Figure 5D. The GCD curves are almost linearly symmetrical and display a slight IR drop, even at a high current density of 10 A/g, which implies good reversibility and conductivity. The specific capacitance of the HPC calculated by equation (3) was about 140 F/g at a current density of 1 A/g. This is attributable to the smaller mesopores and connected flaky structure.

**Figure 6:**
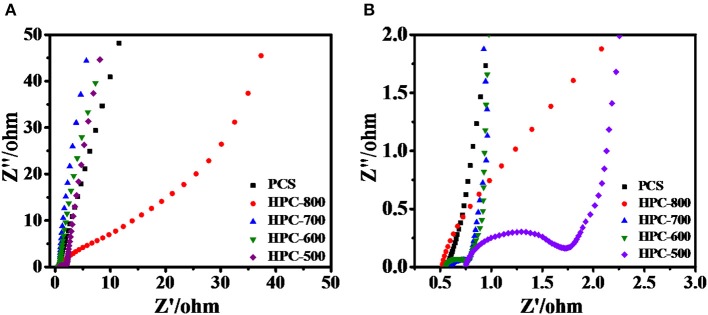
**(A)** Nyquist plots of HPCs and PCS electrodes, and **(B)** enlarged plots of the high frequency region.

The Nyquist plots of HPCs and PCS in a frequency range from 100 kHz to 10 mHz at an open circuit potential in 6 M KOH electrolyte are shown in Figures 6A,B. All samples display a semicircuit-like shape at the high-frequency region, which is ascribed to interface resistance of electrodes and contact resistance between electrodes and collectors. Although the resistance value of HPC-500 was measured to be about 1.5 Ω, the inherent impendence of HPC-600, HPC-700, HPC-800, and PCS was close to 0.21 Ω, 0.22, 0.08, and 0.06 Ω, respectively, which reveals good electronic transport over the regime and good conductivity of prepared samples. At low frequency, other sample curves are nearly vertically linear (~90°), apart from the HPC-800 electrode (~45°), due to over-activation at high temperatures to form destroyed. The slope at low frequency region signifies the degree of ionic penetration from the electrolyte to the surface of the electrode. The larger the slope of the curve, the easier it is for ionic diffusion to occur during the charge/discharge process. The HPC-600 electrode presents the lowest impedance due to having developed a hierarchical pore structure, which implies that HPC-600 could possess better conductivity and excellent ionic diffusion capability (Ding et al., 2018; Han et al., 2019b).

**Figure 7:**
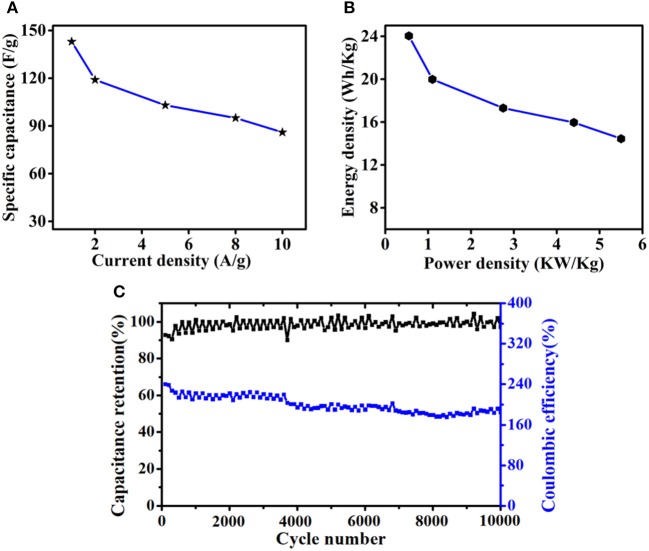
**(A)** The specific capacitance values of an HPC-600 electrode at different scan rates; **(B)** Ragone plot of an HPC-600 electrode; **(C)** cycling performance of an HPC-600 electrode in a symmetric supercapacitor at a current density of 0.5 A/g.

The specific capacitances of HPC-600 at different current densities are shown in Figure 7A. The specific capacitance at different current densities increased with increasing activation time. While the current density was 10 A/g, the specific capacitance could still retain 86 F/g. Energy density and power density are two practical parameters for evaluating the overall energy and power properties of SCs. As shown in Figure 7B, in an aqueous electrolyte system, the HPC-600-based supercapacitor displays a high energy density of 23 Wh/Kg at a power density of 550 W/Kg and remained at 15 Wh/Kg at 5,500 W/Kg. The results can be attributed to the excellent rate capability of HPC-600 and certify that the power density could vary in a wide range without obviously compromising the energy density. Furthermore, cycle stability is an important factor determining whether the material can be used in practical applications. As shown in Figure 7C, HPC-600 as the electrode materials was assembled into a symmetric supercapacitor. The cycle stability of HPC-600 was examined by continuous cycling at 0.5 A/g over 10,000 cycles, and the capacitance retention was 87.5% at 0.5 A/g, demonstrating excellent electrochemical cycling stability for the HPC-600 electrode. In addition, the coulombic efficiency remained 99.9% after 10,000 cycles.

## Conclusions

In summary, a high-performance hierarchical porous carbon for use as supercapacitor electrode materials was successfully achieved by a simple pyrolysis and KOH-activation process. The prepared HPCs derived from Fatsia Japonica show an unusual interconnected hierarchical porous structure composed of meso- and micro- pores despite having a specific surface area of 870.3 m^2^/g. Under optimized conditions, the HPC-600 obtained exhibits a high specific capacitance (140 F/g at a current density of 1 A/g) and also shows excellent cycling stability (87.5% retention after 10,000 cycles). Moreover, the HPC-600-based supercapacitor possesses a power density of about 550 W/Kg and a high energy density of about 23 Wh/Kg, which is about 20% higher than commercial activated carbons. Therefore, it is greatly promising that the sustainable and environmental HPC at activation temperature of 600°C can be used as commercial supercapacitors electrode materials employing Fatsia Japonica, considering the simple large-scale production method and high electrochemical performance.

## Data Availability Statement

All datasets generated for this study are included in the article/supplementary material.

## Author Contributions

SJ provided the idea of the article and corrected the article. HL conducted the experiment and writing. LC assisted in experiments and solved problems on the experiment. FW solved problems on the experiment. GD solved problems on the experiment and corrected data graph. WX provided advice and correction on the revised manuscript. CM provided financial support and instructed the experiment. GZ revised the manuscript and provided final proof. KL and MY provided testing condition.

### Conflict of Interest

The authors declare that the research was conducted in the absence of any commercial or financial relationships that could be construed as a potential conflict of interest.
